# Inorganic polyphosphate regulates neuronal excitability through modulation of voltage-gated channels

**DOI:** 10.1186/1756-6606-7-42

**Published:** 2014-05-31

**Authors:** Stephanie C Stotz, Lucas OM Scott, Christopher Drummond-Main, Yosef Avchalumov, Fernando Girotto, Jörn Davidsen, Maria R Gómez-Gárcia, Jong M Rho, Evgeny V Pavlov, Michael A Colicos

**Affiliations:** 1 Department of Physiology & Pharmacology and the Hotchkiss Brain Institute, University of Calgary, 3330 Hospital Drive NW, Calgary, AB T2N 4N1, Canada; 2 Pediatric Neurology and the Alberta Children’s Hospital Research Institute University of Calgary, Calgary, AB T2N 4N1, Canada; 3 Complexity Science Group, Department of Physics and Astronomy, Faculty of Science, University of Calgary, Calgary, AB T2N 4N1, Canada; 4 Carnegie Institution for Science, Washington, DC 20005, USA; 5 Department of Physiology and Biophysics, Dalhousie University, Halifax, NS B3H 1X5, Canada

**Keywords:** Polyphosphate, Voltage gated channels, Neuroactive compounds, Synaptic vesicles, Synaptic transmission, Neuronal activity, Pain, Platelets, Inflammation

## Abstract

**Background:**

Inorganic polyphosphate (polyP) is a highly charged polyanion capable of interacting with a number of molecular targets. This signaling molecule is released into the extracellular matrix by central astrocytes and by peripheral platelets during inflammation. While the release of polyP is associated with both induction of blood coagulation and astrocyte extracellular signaling, the role of secreted polyP in regulation of neuronal activity remains undefined. Here we test the hypothesis that polyP is an important participant in neuronal signaling. Specifically, we investigate the ability of neurons to release polyP and to induce neuronal firing, and clarify the underlying molecular mechanisms of this process by studying the action of polyP on voltage gated channels.

**Results:**

Using patch clamp techniques, and primary hippocampal and dorsal root ganglion cell cultures, we demonstrate that polyP directly influences neuronal activity, inducing action potential generation in both PNS and CNS neurons. Mechanistically, this is accomplished by shifting the voltage sensitivity of Na_V_ channel activation toward the neuronal resting membrane potential, the block K_V_ channels, and the activation of Ca_V_ channels. Next, using calcium imaging we found that polyP stimulates an increase in neuronal network activity and induces calcium influx in glial cells. Using *in situ* DAPI localization and live imaging, we demonstrate that polyP is naturally present in synaptic regions and is released from the neurons upon depolarization. Finally, using a biochemical assay we demonstrate that polyP is present in synaptosomes and can be released upon their membrane depolarization by the addition of potassium chloride.

**Conclusions:**

We conclude that polyP release leads to increased excitability of the neuronal membrane through the modulation of voltage gated ion channels. Together, our data establishes that polyP could function as excitatory neuromodulator in both the PNS and CNS.

## Background

Inorganic polyphosphate (polyP) is a bioactive polymer of 10 to several 100 orthophosphates linked together by phosphoanhydride bonds. Recent studies have implicated polyP as a regulatory molecule among non-excitable cells [1-4]. Notably, polyP has been shown to play a signaling role in astrocytes where its release facilitates intercellular communication [5] and in stimulated platelets, which release significant amounts of polyP triggering inflammation and plasma clotting [6,7]. Compounds released from stimulated platelets during injury and inflammation are involved in pain signaling mechanisms, and these compounds can directly induce firing of sensory neurons [8].

Here we investigated whether polyP influences neuronal activity. We show that polyP dramatically affects the activity of excitable cells through the modulation of distinct ion channels. Specifically, application of polyP resulted in the rapid generation of high frequency action potentials in both DRG and hippocampal neurons. The neuronal response is due to a hyperpolarizing shift in Na_V_ channel activation, potentiation of Ca_V_ channels, and block of K_V_ channels. As polyP is a neuroactive compound, we propose that its systemic release by platelets contributes to inflammatory pain. Furthermore, we show here that polyP is present in synaptic vesicles using both biochemical and live imaging techniques, and is released from and reloaded into the vesicles by neuronal depolarization. This suggests the possibility that polyP may function as a neurotransmitter under non-pathological conditions. We conclude that polyP functions as a peripheral neuroactive compound and as an endogenous neuromodulator of the CNS.

## Results

### PolyP as a neuroactive compound

**Figure 1:**
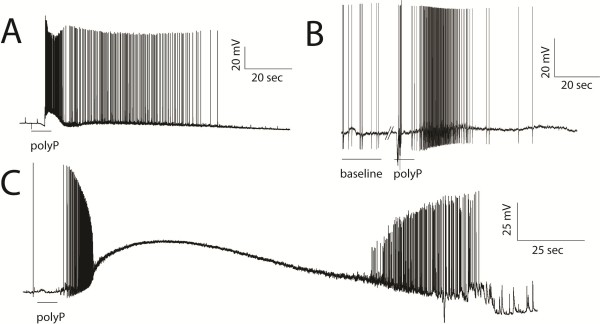
**Neuronal response to polyP.** Both CNS and PNS neurons responded to the application of polyP with a vehement burst of activity. **A)** Representative response of a hippocampal neuron to direct application of polyP (n = 16 of 24), in which rapid, persistent firing is observed. **B)** Representative response of a DRG neuron to polyP. Small nociceptive DRG neurons (<35 microns) responded with rapid firing (n = 24). **C)** Some neurons responded to polyP with a multiphasic response: rapid firing followed by protracted depolarization, during which time firing did not occur, that was restored upon recovery of the membrane potential (8 of 24).

Current-clamp recordings of cultured hippocampal and DRG neurons determined that polyP can functionally stimulate excitable cells. Puff application of medium chain length polyP molecules (60 orthophosphate groups in length on average, see Methods for concentration details) elicited a dramatic increase in AP firing rates from both the CNS (Figure 1A) and PNS (Figure 1B) neurons. Additionally, some hippocampal neurons (n = 8 of 24) had a multi-phasic response to polyP: AP firing frequency increased initially, but ceased as the membrane resting potential depolarized, then AP firing reappeared as the neurons repolarized (Figure 1C). The polyP concentration was chosen based on the reported average polyP concentrations observed in CNS tissue [9,10].

### PolyP application hyperpolarizes the voltage sensitivity of Na_V_ channel activation

**Figure 2:**
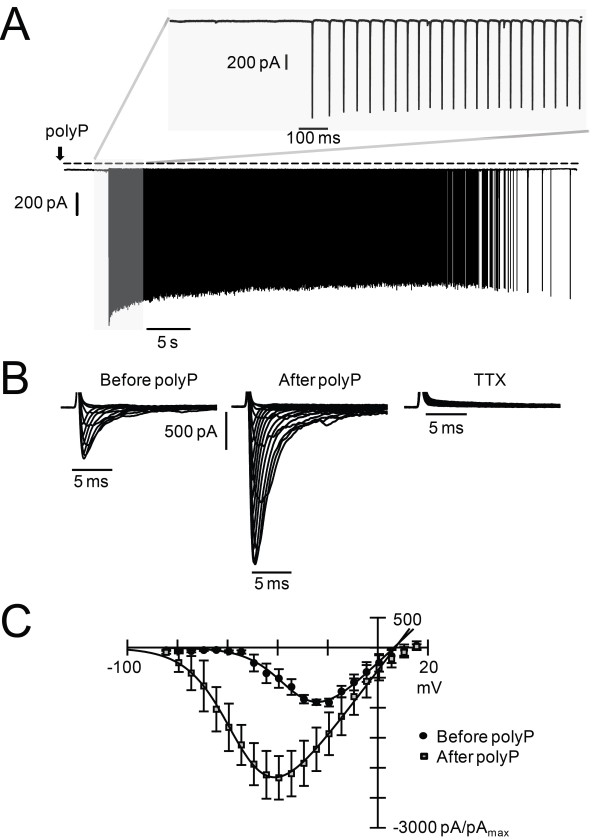
**PolyP application significantly hyperpolarizes Na**_**V **_**channel activation. A)** Holding at -86 mV, polyP application evokes current spikes. Upper panel: expanded time course of activity onset. Lower panel: complete time course for polyP evoked activity. **B)** Representative I_NaV_ traces recorded during a voltage step protocol (see Methods). Before polyP application, I_NaV_ activates at voltages positive to -55 mV. After polyP, I_NaV_ activates at voltages positive to -80 mV. All inward currents were blocked by TTX. **C)** Normalized data plotted as I/Imax versus voltage. Normalized and averaged data (see Methods) were fit with the Boltzmann equation before and after polyP: Vh = -32.5 & -54.3, E_rev_ = 6.4 & 6.9, S = 7.8 & 8.9, G = 39.7 & 55.0, n = 5 & 5, respectively.

Voltage-clamp recordings of cultured hippocampal neurons were conducted to identify the ion channel constituents of the AP whose activity is altered by polyP application. Tetrodotoxin (TTX)-sensitive voltage-gated sodium (Na_V_) channels are highly expressed in most neurons, driving AP generation with the inward flux of a large rapidly activating and rapidly inactivating sodium current. Holding at -86 mV, polyP evoked current spiking activity (Figure 2A, n = 4) completely blocked by TTX (n = 3). Using recording conditions tailored to study Na_V_ channel currents (I_NaV_, see Methods), a step protocol revealed that I_NaV_ activation significantly shifted to hyperpolarized voltages in the presence of polyP (Figure 2B, C and Table 1). A difference of more than 20 mV in the voltage of half activation (V_h_) was calculated from Boltzmann fits of the normalized cumulative data (Figure 2C) before and after polyP application.

**Table 1:** **Na**_**V **_**channel activation properties**

	**Vh**	**Erev**	**S**	**G**	**n**
Before PolyP	-34.7 ± 3.8	8.1 ± 5.7	5.7 ± 0.9	13.3 ± 4.0	5
After PolyP	-55.4 ± 3.9*	5.4 ± 3.8	6.5 ± 1.1	19.8 ± 7.8	5

*p < 0.004, paired Student’s t-test.

### PolyP significantly inhibits I_KV_ and activates I_CaV_

**Figure 3:**
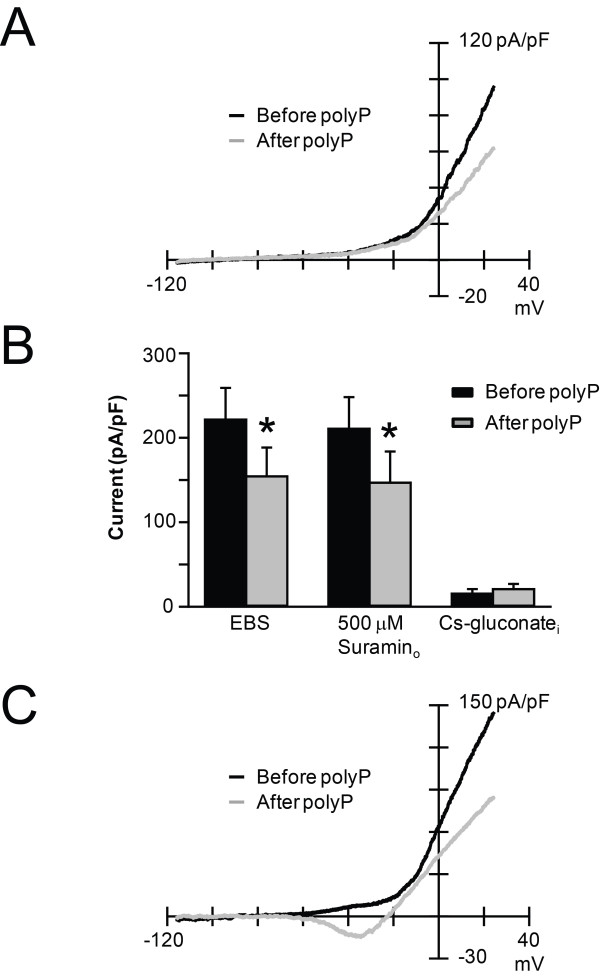
**PolyP significantly inhibits I**_**KV **_**and activates I**_**CaV**_**. A)** Representative I_KV_ traces recorded during a ramp protocol with 0.3 μM TTX in the bath (see Methods). At +24 mV, I_KV_ decreased by 39.5% with polyP application. **B)** Current densities before and after polyP application. 0.3 μM TTX was in the bath. In EBS, I_KV_ decreased by 36.3% ± 5.4 with polyP application (n = 12). With 500 μM suramin in the bath, I_KV_ decreased by 34.5% ± 6.3 with polyP application (n = 6). Using a Cs-gluconate internal solution, I_outward_ increased by 1.7% ± 0.3 with polyP application (n = 6). **C)** Occasionally, I_CaV_ was evoked by polyP application (4 of 7 cells). Shown here are representative current traces where polyP application activated I_CaV_ at -58 mV and blocked I_KV_ (at +24 mV) by 41.8%.

Enhanced excitability of CA1 pyramidal neurons through G protein coupled receptor (GPCR)-evoked inhibition of K_V_ channels is well documented [11-14]. With TTX in the bath, a ramp protocol revealed that polyP addition blocked I_KV_ by 36.3% ± 5.4 (n = 12; Figure 3A & B). However, 500 μM suramin failed to prevent the polyP-induced block of I_KV_ (34.5% ± 6.3, n = 6), suggesting polyP directly blocks I_KV_. To ensure the outward currents are attributable to I_KV_, recordings were conducted with cesium-gluconate internal solutions. Under these conditions, I_outward_ was 15.8 pA/pF ± 5.2 and increased by 1.7% ± 0.3 with polyP application (n = 6). I_outward_ may be attributed to inward chloride flux or non-selective cation channel activity; lanthanum (100 μm), carvacrol (500 μm), APV (50 μm), and CNQX (10 μm) failed to prevent neuronal excitation. The functional expression of low threshold voltage activated calcium (Ca_V_) channels, both T- and R-type, in hippocampal neurons has been well described [15-19]. With TTX in the bath, polyP application evoked low threshold voltage activated calcium currents (I_CaV_) in 4 of 7 cells. I_CaV_ activated at -44 mV ± 6.5 (n = 4), and was blocked by 300 μM NiCl.

### PolyP stimulates neurons and glia in hippocampal co-cultures

**Figure 4:**
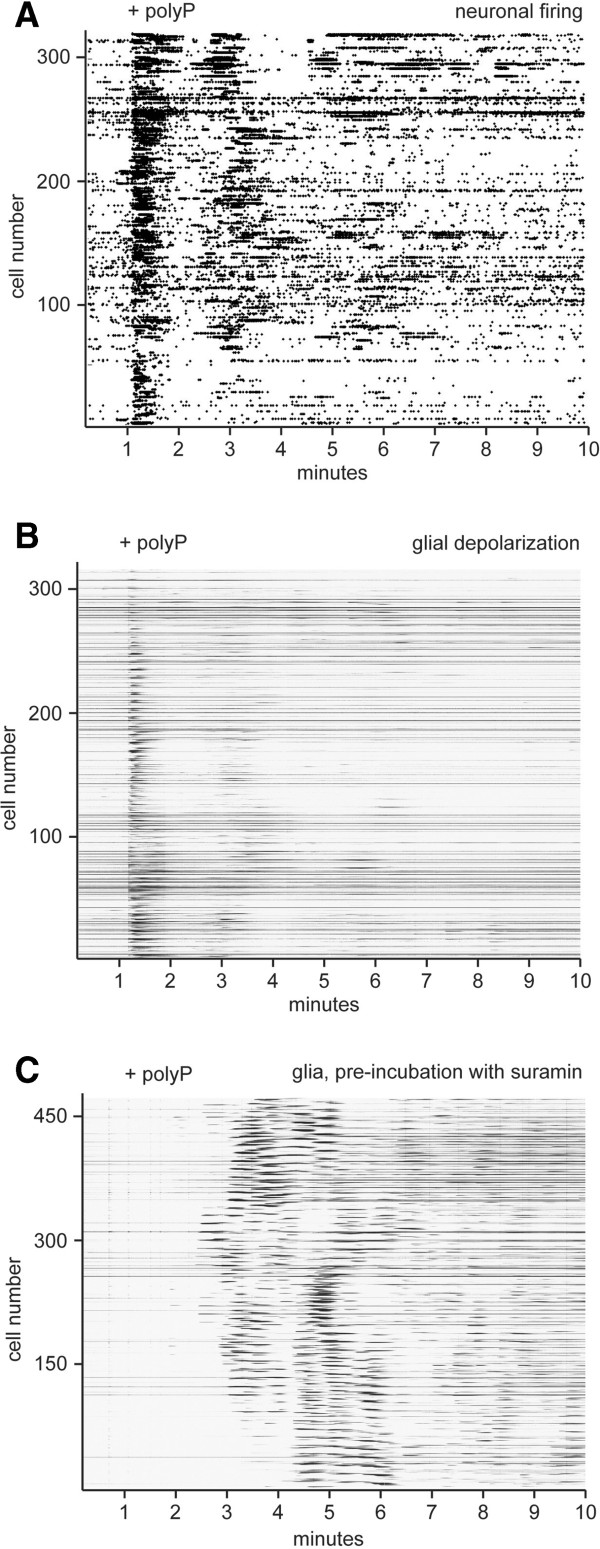
**PolyP stimulates neurons and glia in hippocampal co-cultures. A)** Addition of polyP causes immediate firing of neurons, with many of the cells having a secondary burst a few minutes later. **B)** PolyP evoked glia depolarization from the same experiment as A), showing concurrent activation of the glia. **C)** Glia activity corresponding to the neuronal firing in B), showing the presence of suramin resulted in delayed glial activity evoked by polyP.

To confirm our electrophysiology data and extend our analysis to glia cells, we assessed the response of dissociated hippocampal neuronal/glia co-cultures to the addition of polyP using high spatial and temporal resolution calcium imaging. Our analysis software separates the slower responses attributable to depolarization of the underlying astrocyte layer from neuronal action potentials, producing activity “fingerprints” of >300 neurons simultaneously (see Methods for experimental details). Figure 4A shows the response of the neurons to polyP, where most neurons responded immediately with a burst of activity and some but not all had a secondary burst a few minutes later (Figure 4A), consistent with the patterns observed in Figure 1B. In Figure 4B we show the underlying glial activity patterns corresponding to the neuronal pattern seen in the experiment in Figure 4A. PolyP evokes glial calcium signals during polyP addition in synchrony with neuronal activity, potentially driven by glutamate release from the actively firing neurons. In astrocytes, polyP binding to P2Y_1_ receptors stimulates Gq-coupled calcium release from internal stores [5]; here we show that suramin prevents the initial burst of glial activity (Figure 4C). However, a few minutes after polyP addition glial activity increased, potentially in response to neuronal activity.

### DAPI stains polyP in distinct punta along axon-like projections

**Figure 5:**
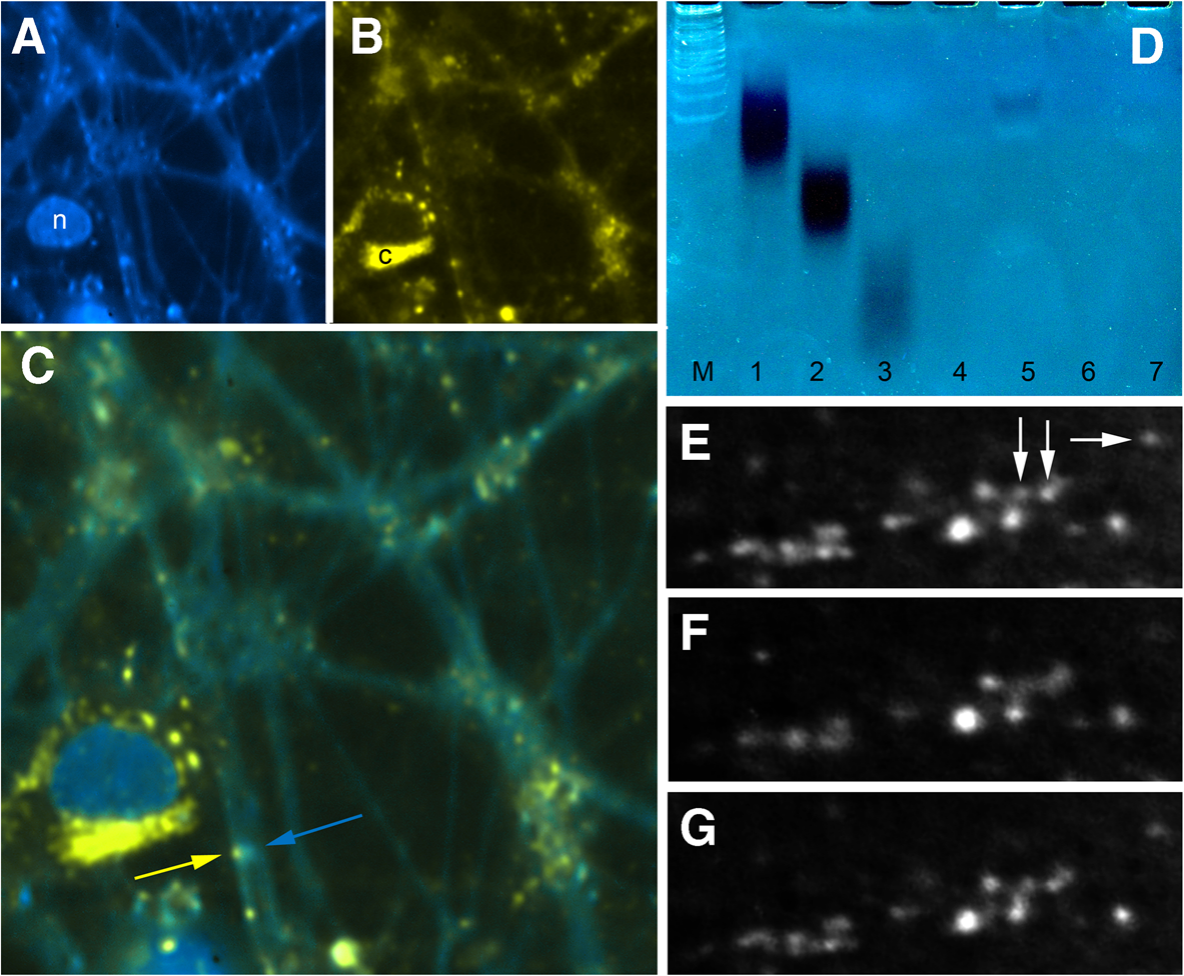
**DAPI stains polyP in distinct punta along axon-like projections. A)** At 456 nm (emission) DAPI stains nucleic acids primarily in the nucleus but also in puncta. **B)** At 560 nm (emission) DAPI stains polyP. PolyP is in the perinuclear space and cytoplasm of the cell body, and is found in distinct puncta along axon-like projections. **C)** Overlay of A&B, showing polyP puncta on axon-like filaments across from dendritic structures. **D)** Gel electrophoreses demonstrating presence of and release from synaptic vesicles of polyP. Lanes: M - DNA marker; 1 - long polyP (average 130 chain length); 2 - medium polyP (average 60 chain length); 3 - short polyP (average 15 chain length); 4 - Synaptic vesicles + 70 mM KCl; 5 - Synaptic vesicles alone; 6 - Synaptic vesicles + 70 mM KCl + phosphatase (sample from lane 4); 7 - Synaptic vesicles + phosphatase (sample from lane 5). 70 mM KCl evoked vesicular depolarization. Phosphatase digested polyP. Live imaging of *in vitro* polyP release from and uptake into distinct puncta - **E)** T = 0. The arrows indicate distinct puncta to follow through **F** &**G**. **F)** Following a 10 second stimulation at 60Hz, a subpopulation (16% +/-5, SEM, n = 6) of polyP puncta disappear. Adjacent polyP puncta remain, indicating that there are no confounding focal plane shifts or spatial movements. **G)** 30 seconds later the diminished puncta have begun to refill with polyP.

PolyP is a gliotransmitter, released by astrocytes to signal neighboring astrocytes [5]. Here we demonstrate that polyP is also released and taken up by neuronal synapses. PolyP can be imaged in live cells using DAPI stain and collecting emission wavelengths at 560 nm [1,20-22]. In our hippocampal cultures polyP is found in distinct puncta along axon-like projections and in the cell body perinuclear space and cytoplasm (Figure 5A). In contrast, at 456 nm emission, DAPI stains nuclear DNA and dendritic nucleic acids (Figure 5B). Overlap of the two emission signals clearly shows the differential compartmentalization of polyP versus nucleic acids (Figure 5C). Photoconductive stimulation can induce neuronal cultures grown on silicon wafers to fire in a high frequency, physiological manner [23]. Before stimulation, multiple polyP positive puncta are visible along axon-like projections (Figure 5E). Immediately following stimulation a subpopulation of the puncta disappear (Figure 5F), suggesting that the polyP is released concurrent with neuronal activity. As not all puncta vanish (~16% +/-5 of total puncta remain, n = 6 experiments), polyP may be in vesicles and other compartments outside of the readily released pool. The remaining puncta provide corroborative evidence that there was no shift in focal plane during the imaging. Interestingly, puncta reappeared within 30 seconds of the firing event (Figure 5G), suggesting that the polyP is rapidly replenished. Together, these data suggest polyP is can be released and replenished into neuronal puncta, similar to other neurotransmitters.

As DAPI fails to label polyP in fixed cells, co-staining with cellular/synaptic markers was not possible. However, polyP isolated from synaptosomes can be labeled with DAPI (Figure 5D lane 5). Compared to synthetic polyP of varying lengths (Figure 5D lanes 1–3), synaptically isolated polyP ran parallel to long polyP (~130 polymer chain length). An *in vitro* enzymatic assay (see methods) indicated that the synaptic vesicle fraction contained ~35 pmol/mg of protein. Phosphatase treatment completely hydrolyzed synaptic polyP (Figure 5D, lane 6 and 7). Importantly, synaptosomal release of polyP can be triggered by KCl-evoked membrane depolarization as evidenced by the reduction of the polyP band density (Figure 5D, lane 4). This supports the possibility that similar release of polyP can occur in response to membrane depolarization in the intact neurons.

## Discussion

PolyP stimulates neuronal activity by modulating several key components of the action potential. Our results show that polyP blocks K_V_ channels, and potentiates Na_V_ and Ca_V_ channel activity. K_V_ channels contribute to the maintenance of the neuronal resting membrane potential. When K_V_7 in particular, is inhibited, depolarization follows [24], and spontaneous CA1 pyramidal neuron firing is induced [14], presumably as the threshold for Na_V_ channel activation is reached. PolyP further promotes AP generation by shifting Na_V_ channel voltage sensitivity so that less depolarization is required to trigger Na_V_ channel activation. PolyP also magnifies the entry of calcium through Ca_V_ channels, potentially amplifying downstream intracellular signaling pathways. The concerted effects of polyP on neuronal ion channels may promote normal AP generation, while excessive release of polyP may contribute to hyperexcitability disorders such as epilepsy.

In astrocytes, polyP activation of the P2Y1 receptor Gq signaling pathway underlies glial communication [5]. The polyP effect we report on neuronal ion channels differs significantly from the well characterized effect of P2Y GPCR activity on these same channels [25]. In particular, nucleotides inhibit I_CaV_ through the activation of P2Y1 receptors [26], while polyP potentiates I_CaV._ Nucleotides inhibit the TTX-sensitive I_NaV_[27], as does GPCR activation of PKA/PKC [28]; polyP significantly hyperpolarizes activation of I_NaV_. P2Y1 receptors activate hippocampal neuron calcium-dependent K^+^ channels [29], however, polyP did not elicit this activity. Nucleotides inhibit I_KV_ through P2Y1 receptors [24], an effect mimicked by polyP. In our calcium imaging experiments, suramin did prevent polyP-evoked signaling in glia. However, suramin failed to prevent neuronal polyP I_KV_ inhibition, suggesting the GPCR pathway is not involved. Rather, we suggest that polyP effects neuronal ion channel gating properties by altering the local surface charge. Long chain polyP is high in negative charges and may increase the density of fixed negative charges surrounding the channels [30]. As a result polyP would create an environment promoting Na_V_ and Ca_V_ channel activation. We predict that the K_V_ channels are directly blocked by polyP. The ability of polyP to simultaneously modulate the activity of a range of channels further supports the interpretation that its activity is linked to the global electrostatic change of the channels environment rather than its binding to specific sites. A parallel can be made to a recent study which proposes that polyP acts as a chaperone, simultaneously interacting with a number of proteins [31]. In contrast, polyP modulation of TRPM8 channels is attributed to direct ionic interactions [3,32].

We found that polyP accumulates in synaptic vesicle-like structures under physiological conditions. Upon depolarization, and in response to a burst of neuronal activity, polyP is released into the extracellular milieu where it will stimulate further neuronal and glial communication. Astrocytes are also a source of polyP [5]. Abramov’s research group recently reported that less that 3% of neurons responded to 50 μM polyP ([5]; calcium imaging experiments). For our electrophysiology experiments, we chose to apply polyP using a puffer pipette. The technique mimics synaptic release more closely; however the targeted neurons see a higher concentration of the applied agonist before it diffuses into the bath. 100% of neurons tested responded to polyP.

PolyP regulates many critical cellular processes including mitochondrial ion transport and respiratory chain activity [33,1], and is a constituent of the normal physiological environment. However, pathological conditions can lead to a dramatic increase in polyP concentrations. During inflammation polyP is released from activated platelets [34,6], stimulating blood coagulation [7]. Given its activity on sensory DRG neurons, polyP is expected to elicit a pain response. Supporting this hypothesis, peripheral sensory TRP channel activity is dependent on polyP. Specifically, normal activity of TRPM8 requires the presence of polyP as a structural component of the channel [3] while TRPA1 channels rely on polyP as a cofactor regulating their activity [35]. Centrally, epileptic seizure activity could be expected with a dramatic increase in polyP. It will be interesting to determine if polyP concentrations rise under conditions of inflammation, stroke, seizure, or ischemia, contributing to global neuronal changes.

## Conclusions

We conclude that inorganic polyphosphate is a previously unrecognized modulator of neuronal Na_V_, Ca_V_, and K_V_ channels, potentially playing an important role in normal neuronal synaptic transmission as well as pain and other neuropathological conditions.

## Methods

### Neuronal cultures

Hippocampal co-cultures (containing both neurons and glia) were prepared as previously described [36] and used for the electrophysiology, network activity and polyP imaging experiments. Briefly, hippocampi were dissected from P0 (newborn) Sprague Dawley rat pups, and neurons and glia were dissociated and plated together on pretreated silicon wafers [23]. Cultures were maintained in Eagle’s Basal Media (BME, GIBCO-Invitrogen) supplemented with B-27, penicillin, streptomycin and L-glutamine. Cultures are maintained with 4% Fetal Bovine Serum (FBS) for the first week to aid in establishment of the cultures; FBS is subsequently reduced to restrict growth of the astrocytes. Functional synapses form after a few days in culture, and robust spontaneous network activity could be observed after approximately 2 weeks. Dorsal root ganglion (DRG) neurons were cultured as previously described [37].

### DAPI staining of neuronal cultures

2 weeks old hippocampal neuronal cultures were stained with DAPI (4′,6-diamidino-2-phenylindole) at a final concentration of 0.1 μg/ml in extracellular bath solution (135 mM NaCl, 2 mM MgCl_2_, 3 mM CaCl_2_, 5 mM KCl, 5 mM HEPES, and 10 mM Glucose, pH 7.4 with TEA-OH and 320 mOSM with D-sorbitol) for 5 minutes. Cultures were rinsed briefly and imaged in the 560 nm (emission) range to detect polyP, and at 456 nm to detect nucleic acids. For the polyP channel, color images of the emission band were taken with a Nikon D3 camera, and then the specific wavelength further refined with color band filtering of the RGB image. Separated images were then recombined for the static image presentation (Figure 5A-C) using Photoshop.Live imaging of release of polyP from synapses (Figure 5E-G) was performed on the same cultures, using the same DAPI-polyP filter set, without the post color band processing.

### Synaptosome and synaptic vesicle isolation

Synaptosomes and synaptic vesicles were isolated from cortical tissue of one adult Sprague Dawley rat. A synaptic vesicles isolation kit (Sigma, SV0100) was used to prepare an enriched synaptic vesicle fraction. The synaptosomal pellet was resuspended in lysis buffer and incubated on ice for 45 minutes, followed by centrifugation at 20,000 g for 20 minutes. Synaptic vesicles were collected as a pellet by final centrifugation of resultant supernatant at 70,000 g for 45 minutes and resuspended in storage buffer. Isolated vesicles were frozen and further processed for polyP extraction and concentration determination.

### Reagents

Purified polyP of either 130 (long), 60 (medium) or short (15) average chain length was a kind gift of Toshikazu Shiba (RegeneTiss). Direct application experiments used medium length chain polyP. Stock solutions of 0.5 M were diluted 1:10 and puff applied (2 μl) to the recording field for a final bath concentration of 50 μM. Channel blockers included lanthanum (L4131 Sigma), carvacrol (W224502 Aldrich), APV ((2R)-amino-5-phosphonopentanoate, Sigma A8054), CNQX (6-cyano-7-nitroquinoxaline-2,3-dione, Sigma C239), suramin (S2671 Sigma), PPADS (pyridoxalphosphate-6-azophenyl-2′,4′-disulfonic acid, Sigma P178), TTX (tetrodotoxin, Sigma T5651), TEA-Cl (tetraethyl ammonium chloride, Sigma T2265), TEA-OH (tetraethylammonium hydroxide, Sigma 86633) and NiCl_2_ (nickel chloride, Aldrich 339350).

### PolyP extraction and concentration determination

PolyP was extracted from synaptic vesicle preparation using chloroform-methanol extraction protocol. 100 μL of synaptic vesicles in storage buffer were incubated with 60 μL of methanol for 20 minutes followed by the addition of 120 μL of chloroform. PolyP was collected from the aqueous (upper) phase of the resultant mixture. Collected sample was concentrated to 40 μL using a Speed Vac and run on a PAGE gel (15%). The running buffer (TBE) contained (mM) 90 Tris, 90 borate, 2 EDTA, pH 8.3. Gels were run at 100 V for 1 hour and incubated in a fixative solution containing 25% methanol and 5% glycerol at pH = 8.0. PolyP was visualized using negative staining with DAPI as previously described [38]. Briefly, gels in fixative solution were incubated with 2 μg/ml of DAPI for 30 min and then exposed to 365 nm light using a UV transilluminator for 20–30 min to induce photobleaching. DAPI bound to polyP produced strong photobleaching which resulted in the appearance of dark bands on the gel. Samples were treated with calf alkaline phosphatase (10 U/ml; Sigma).

PolyP was colorimetrically quantified as the amount of orthophosphate residues (Pi) released upon sample treatment with recombinant exopolyphosphatase (PPX; from yeast Saccharomyces cerevisiae). Sample was mixed with 100 mM Tris–HCl, pH 7.5, 50 mM ammonium acetate, 5 mM magnesium acetate, and PPX in excess to ensure complete polyP hydrolysis. 50 μl of the processed sample was then mixed with 500 μl of 2.5% solution of ammonium molybdate prepared in 5 N sulfuric acid and 50 μl Fiske-Subbarow reagent (Sigma), with the total volume adjusted to 1 ml with water. The optical density of the solution at 650 nm was compared with Pi standards (Sigma) to determine the sample quantity of Pi [21].

### Electrophysiology

Current and voltage clamp recordings were obtained from primary culture hippocampal and DRG neurons. Small (nociceptive) DRG neurons were used for electrophysiological recording after more than 24 hours in culture to allow adequate extension of processes. Hippocampal neurons were cultured for 2 weeks to allow extensive synaptic networks to develop before recordings were made, with the exception of I_NaV_ records. I_NaV_ recordings were obtained from hippocampal neurons cultured 3 days and treated for 5 minutes with PBS versene (Invitrogen) to mitigate space clamp issues arising from extensive neuronal processes. For I_NaV_ recordings the external media contained (in mM): 50 NaCl, 100 TEA-Cl, 5 MgCl_2_, 5 KCl, 5 HEPES, and 10 Glucose, pH 7.4 with NaOH and 320 mOSM with D-sorbitol. For all other recordings and imaging experiments the external bath solution (EBS) contained (in mM): 135 NaCl, 2 MgCl_2_, 3 CaCl_2_, 5 KCl, 5 HEPES, and 10 Glucose, pH 7.4 with TEA-OH and 320 mOSM with D-sorbitol. Suramin (500 μM) or TTX (0.3 μM) were mixed in EBS to their final concentrations before replacing the hippocampal culture media. DRG internal pipette solution contained (in mM): 100 K-gluconate, 1.7 KCl, 0.6 EGTA, 5 MgCl_2_, 10 HEPES, 3 ATP and 0.6 GTP, pH 7.25 with KOH and 300 mOsm with glucose. Hippocampal internal pipette solution contained (in mM): 130 K-gluconate, 0.1 EGTA, 0.3 MgCl_2_, 7 NaCl, 10 HEPES, 3 ATP and 0.6 GTP, pH 7.25 with KOH and 300 mOsm with glucose. Cesium gluconate (Cs-gluconate) internal pipette solution contained (in mM): 130 Cs-gluconate, 0.1 EGTA, 0.3 MgCl_2_, 7 NaCl, 10 HEPES, 3 ATP and 0.6 GTP, pH 7.25 with CsOH and 300 mOsm with glucose.

Whole-cell patch clamp recordings from DRGs and hippocampal neurons were made using a Multiclamp 700B amplifier, a Digidata 1440A digital-to-analog converter, and pClamp10 software (Molecular Devices, Palo Alto, CA). Current-clamp recordings were performed by switching to current-clamp mode after a stable whole cell configuration was formed in voltage clamp mode. A membrane potential of at least of -40 mV was obtained from cells used in the statistical analysis. In a few cases, depolarizing pulses of 10 to 20 pA, lasting about 1–2 s, caused local increases in membrane potential to -30 mV. The experiments were performed at room temperature (21-23°C). During voltage-clamp recordings, capacitance and series resistance were compensated by 75-85%. Ramp protocol: from a holding potential (HP) of -70 mV, voltage ramps from -100 to +40 mV (400 ms in duration, with a 40 ms delay at -100 and +40 mV) were applied every 2 seconds. Data were low-pass filtered at 2 kHz and digitized at 10 kHz. Step protocol: from a HP of -100 mV, 30 ms voltage steps in 5 mV increments from -70 to +20 mV were applied every 2 seconds. Data were low-pass filtered at 6 kHz and digitized at 50 kHz. Data are presented with liquid junction potentials (LJP) corrected for.

Data analysis of I_NaV_: Peak I_NaV_ from the step protocol was plotted as a function of the test potential (LJP corrected) to generate current–voltage relations (I-V). For the mean I-V (Figure 3C), paired data were normalized to the I_peak_ before polyP application and averaged. Individual (Table 1) and mean I-V relations (Figure 3C) were fit with the Boltzmann equation, where I_peak_ = (V - E_rev_) G (1/1 + exp (V_a,1/2_ - V)/S)), and E_rev_ is the reversal potential, V_a,1/2_ is the half-activation potential, G is the maximum slope conductance, and S is the slope factor that is inversely proportional to the effective gating charge.

#### ***Data analysis of I***_***KV***_ ***and I***_***CaV***_

Currents recorded during ramp protocols were normalized by cell capacitance measurements for the comparison of current densities.

#### ***Statistical analysis***

A paired Student’s t-test was used to determine if significant differences resulted from polyP application. Results are reported in the figures.

### Calcium imaging and recording of network activity

18–21 DIV neuronal cultures for the network activity analysis were prepared as described above. To capture neuronal and glial activity, cultures were first loaded with the calcium dye Fluo4-AM by incubating the silicon wafer in 500 μl of recording solution and 2 μg of Fluo-4 at 37°C for 30 minutes prior to optical recording. Wide field images of neuronal activity were made at 15 frames per second using an Olympus BX60 microscope with a 10× water immersion objective and a Watec WAT-120 N camera. Individual neuronal cell bodies in the images were first identified from an averaged frame spanning the time course of the recording, based on their morphology. Neurons were numbered and their activity traces extracted by calculating the mean grey value in a region of interest (ROI) encompassing the cell body. Their identity as neurons was confirmed by the criterion that action potentials are detected, as determined by a sharp (less than 15 ms) rise in internal calcium. We then removed the depolarization patterns of the neurons from the underlying glia calcium signal, and segregated the field of view into a grid array. Changes in these surrounding regions are taken as glial depolarizations, again confirmed by their gradual increase and decrease in comparison to the more rapid speed of a neuronal firing event.

Once the neuronal and glial movies were split, each was processed by custom written routines in Matlab. For the neuronal analysis, activity fingerprints were made that display the neuron index number vertically, and the point in time that a firing event occurred horizontally. For the glial depolarizations, each grid reference point was treated like a cell, and displayed in a similar manner to the neurons. This provided a general correspondence of the physical region of the glial activity with the reference point of the neurons. In all experiments light intensity was kept to the minimum in order to avoid photobleaching. 3 separate rounds of hippocampal cultures were used for imaging experiments.

### Use of animals

Newborn rat pups (not gender identified) for primary neuronal cultures were obtained from Sprague Dawley dams, purchased from Charles River Laboratories International, Inc. All experimental protocols were approved by the University of Calgary Conjoint Faculties Research Ethics Board, and performed under protocol #M5083.

### Statistics

No parametric statistical analysis of imaging was performed for this manuscript as all observations were binary events (e.g. if bursting occurred or not, if synaptic staining disappeared or not). n values represent the number of times an experiment was repeated and the phenomena was observed.

## Competing interests

The authors declare no competing financial or non-financial interests.

## Authors’ contributions

SS performed the voltage gated channel analysis and in part wrote the manuscript. LS performed the biochemical analysis of polyP. LS and SS produced the cultures. SS, CDM and YA performed electrophysiology. FG coded and ran the activity analysis. JD contributed to the activity analysis. MRGG quantified the synaptic vesicle polyP amounts. JR provided guidance on the electrophyiology. EP initiated the study and performed the biochemistry, and in part wrote the manuscript. MC performed the *in vitro* live imaging, electrophysiology and in part wrote the manuscript. All authors read and approved the final manuscript.
